# Gene Mutations in Gastrointestinal Stromal Tumors: Advances in Treatment and Mechanism Research

**DOI:** 10.1055/s-0044-1789204

**Published:** 2024-08-22

**Authors:** Lei Cao, Wencong Tian, Yongjie Zhao, Peng Song, Jia Zhao, Chuntao Wang, Yanhong Liu, Hong Fang, Xingqiang Liu

**Affiliations:** 1 Department of General Surgery, Tianjin Union Medical Center, Tianjin, People's Republic of China; 2 Tianjin Key Laboratory of General Surgery in Construction, Tianjin Union Medical Center, Tianjin, People's Republic of China

**Keywords:** GISTs, gene mutation, molecular mechanism, targeted therapies, drug resistance

## Abstract

Although gastrointestinal stromal tumors (GISTs) has been reported in patients of all ages, its diagnosis is more common in elders. The two most common types of mutation, receptor tyrosine kinase (KIT) and platelet-derived growth factor receptor a (PDGFRA) mutations, hold about 75 and 15% of GISTs cases, respectively. Tumors without KIT or PDGFRA mutations are known as wild type (WT)-GISTs, which takes up for 15% of all cases. WT-GISTs have other genetic alterations, including mutations of the succinate dehydrogenase and serine–threonine protein kinase BRAF and neurofibromatosis type 1. Other GISTs without any of the above genetic mutations are named “quadruple WT” GISTs. More types of rare mutations are being reported. These mutations or gene fusions were initially thought to be mutually exclusive in primary GISTs, but recently it has been reported that some of these rare mutations coexist with KIT or PDGFRA mutations. The treatment and management differ according to molecular subtypes of GISTs. Especially for patients with late-stage tumors, developing a personalized chemotherapy regimen based on mutation status is of great help to improve patient survival and quality of life. At present, imatinib mesylate is an effective first-line drug for the treatment of unresectable or metastatic recurrent GISTs, but how to overcome drug resistance is still an important clinical problem. The effectiveness of other drugs is being further evaluated. The progress in the study of relevant mechanisms also provides the possibility to develop new targets or new drugs.

## Introduction

Gastrointestinal stromal tumors (GISTs) are the most common mesenchymal neoplasms, 1 2 accounting for only about 1% of primary gastrointestinal malignancies. 3 GISTs originate from the interstitial cells of Cajal (ICCs) or their precursors, which are located within the muscle layers of the alimentary tract and function as pacemaker cells. 4 Among them, approximately 60 to 65% being localized in the stomach and approximately 25 to 30% in the small intestine. 5 6 A small number of cases have also been reported in rectum, colon, esophagus, and other sites. 7 8 Research survey shows that GIST occurring in children and young patients (<30 years of age) arise mostly at gastric sites. 9 However, GISTs are also reported to be found in extra-gastrointestinal sites such as omentum or retroperitoneum. 10

The incidence of GISTs is about 12 cases per 10 ^6^ individuals per year in most countries, with differences between regions and over time. 7 11 American studies have found that Asian/Pacific Islanders Black people got a higher incidence than White people. 12 In addition, GISTs display approximately equal distribution in gender. 3 The common clinical symptoms of GISTs includes bleeding, pain and/or obstruction, and the tumor size may range in diameter from a few millimeters to more than 30 cm, among which, <1 or 1 to 2 cm are frequently termed micro-GISTs or mini-GISTs, respectively. Although GISTs have been reported in patients of all ages, they are more frequently diagnosed in older patients, with an average age at diagnosis ranges from 62 to 75 years and a peak incidence in the 8th decade of life. 7 Less than 10% of patients are younger than 40 years, whereas they are quite rare in children and young adults. 13 Moreover, micro- and mini-GISTs have been identified in up to 30% of elderly individuals. 6 14

The two most common types of mutation, receptor tyrosine kinase (KIT) and platelet-derived growth factor receptor a (PDGFRA) mutations, hold about 75 and 15% of GISTs cases, respectively. 15 16 17 Tumors without KIT or PDGFRA mutations are known as wild type (WT)-GISTs, which takes up for 15% of all cases. 18 19 WT-GISTs have other genetic alterations, including mutations of the succinate dehydrogenase (SDH) and mutations in Ras family genes: serine–threonine protein kinase BRAF and neurofibromatosis type 1 (NF1). 20 21 The other GISTs without mutations in any of the previous genes have been named as “quadruple WT” GISTs, 18 in which additional molecular alteration and very rare gene fusions have been reported. 22 23 These mutations or gene fusions are considered to be mutually exclusive in primary GIST, but coexistence of some of these rare mutations with KIT or PDGFRA mutations has been reported recently. 7 24

The therapeutical management for GISTs is different depending on molecular subtype, especially for the patients with advanced GISTs, assessment of mutational status is necessary for developing a personalized chemotherapy plan to improve the patients' survival and quality of life. Here, we focuses on four major genetic alterations of GISTs, update various variants and their core regulatory network, summarize and update the treatment options and research progress for these types of tumors, and introduce the key problems encountered in related research and therapy.

## KIT Mutations

### Types of Genetic Mutation

Roughly 75% of GIST cases present activating mutations in KIT gene. 16 25 The available data suggest that GIST with KIT mutations have an incidence close to 8 cases per 10 ^6^ individuals per year in most regions, GIST with KIT mutations are most common in individuals >18 years of age. 7 9 KIT mutations are also found in micro-GIST and mini-GIST, as well as in familial GIST resulting from germline mutations in these genes. These patients have diffuse hyperplasia of ICC and multiple benign small GIST. 7 14

KIT gene located on chromosome 4q12 and contains 976 amino acids, encodes a transmembrane protein belonging to type III receptor tyrosine kinases. 3 26 From a structural perspective, KIT is constitute of five extracellular immunoglobulin-like domains (D1–D5), a single transmembrane helix, a cytosolic juxtamembrane (JM) domain, two kinase domains (TK1 and TK2) and a C-terminal tail. 6 7 Domains of D1–D3 and D4–D5 are responsible of ligand binding and receptor dimerization, respectively, and TK1 (including ATP-binding pocket, ABP) and TK2 (including activation loop, A-loop) is separated by a kinase insert domain. 6 7 The most prevalent mutations including deletions, deletion–insertions (indels), insertions and missense mutations that occur mostly in exons 8, 9, 11, 13/14, and 17/18, among them, mutations in exon 11 are the most frequent with a percentage of 61 to 71%. 27 28 Both exon 8 and exon 9 are located in the D5 domain, exon 11 falls in the JM domain, exons 13/14, and exons 17/18, respectively, corresponds to the TK1 ABP and TK2 A-loop. 6

GIST with KIT exon 11 mutations can be observed at any anatomical site in the gastrointestinal tract. 7 29 The JM domain usually perform the function of stabilizing the inactive conformation of KIT receptors and inhibiting dimerization. Mutations resulting in loss of function of the JM domain induce dimerization and autophosphorylation, 7 30 leading to sustained autonomic activation, uncontrolled proliferation, and inhibition of apoptosis. Mutations in the JM domain are mostly caused by in-frame deletions in codons Gln550 and Glu560, known as a hot spot region. 31 Besides, deletion of W557 and/or K558 has been reported in 28% of all GISTs and is associated with high-risk tumors due to clinicopathological features. 7 31

According to statistics, 20 to 25% of GIST cases bearing KIT exon 9 mutations, 6 32 among which the most common mutation is repeated insertion of Ala502 and Tyr503, 7 31 KIT protein with this AY duplication has a kinase conformation similar to that of wild-type KIT for SCF binding. These types of tumor usually arise in the small intestine, colon, or rectum and often possess more aggressive characteristics. 20 30 Additional rare mutations have also been described. 6 33 Exon 9-mutated GISTs have been reported to tend more to metastasize to the peritoneum than to the liver in comparison with WT-GISTs and Exon 11-mutated GISTs. 34 Additionally, mRNA level of stem cell factor (SCF) is markedly upregulated in exon 9-mutated tumors, leading to an autocrine proliferative loop, along with overexpressed mRNAs from genes involved in the WNT pathway, 6 which has been shown to contribute to GIST malignancy. 35

Other less frequent KIT spots are in exon 13, 17, and 8 and occur in approximately 1 to 2% of KIT-GISTs. 30 Tumors with exon 13 mutations are most often found in the small intestine, usually have a spindle cell morphology, are slightly larger, and are more aggressive tumors than other types of GIST. Regarding exon 17, the 70% of mutations is Asn822Tyr, these tumors arise frequently in the small intestine and usually present a spindle cell morphology. 2 30 Asp820Tyr mutation is also detected previously, and Arima et al present the case of a patient with multiple GISTs with a novel germline KIT gene mutation (Asp820Gly) in exon 17. 2 Furthermore, mutations in exon 8 occur rarely in GIST. These tumors are associated with a malignant phenotype and multiple peritoneum metastasis. 36 A 53-year-old Japanese patient was reported to have a deletion of Asp419 at exon 8; this mutation caused the receptor to activate continuously. 37 Subsequently, cases of GISTs with substitution of ThrTyrAsp (417–419) to Tyr (TYD417-419Y) were found in another two cases. 37 40 However, the number of GIST cases with exon 8 mutations appears to be very small. 37 40 In an analysis of 48 GIST tissue samples, 21 different variants were detected in the KIT gene, 8 of which were novel changes, and mutations in exon 11 were identified 28 cases (58.3%). 41

Different KIT mutations have different effects on the protein inactive and active structures, dimerization affinity, and cellular localization. KIT mutations arising in exon 11 relieve the autoinhibitory constraint of the JM domain and, therefore, lead to constitutively activated ligand-independent KIT variants. 6 42 These variants have negligible membrane localization undergo constitutive ubiquitination, internalization, and degradation. 42 While KIT mutations in exon 9 lead to oncogenic KIT variants with increased dimerization affinity and elevated basal TK activity, they can respond to SCF stimulation at much lower concentrations. 43 These KIT variants maintain a partial localization to the cell membrane; undergo ligand-induced ubiquitination, internalization, and degradation; and have a prolonged half-life in unstimulated cells. 38 39 42 Instead of transferring to the cell membrane, KIT variants retained within the endoplasmic reticulum and Golgi in a constantly activated state. 6 44 According to a study by Obata et al, Golgi retention of KIT is associated with activation of PLCγ2-PKD2-PI4KIIIβ (phospholipase Cγ2-protein kinase D2-phosphatidylinositol 4-kinase IIIβ) pathway in GISTs. 45 KIT mutations are early events for the development of GIST from ICC, meaning KIT necessitates the survival of GIST cells. 46 Feedback loops result in inhibition of SCF-induced autophosphorylation and in SCF-induced ubiquitination, internalization, and degradation of KIT. 6 47 After activation, KIT mediates its effects on cell growth, differentiation, and apoptosis and also promotes tumorigenesis and malignant progression, 3 7 through regulating multiple downstream signal pathways such as PI3K-AKT pathway, JAK-STAT pathway, and mitogen-activated protein kinase (MAPK) pathway. 3 6 7 48 49 However, KIT mutations are considered to be not sufficient for the neoplastic transformation of ICC into GIST. Subsequent changes of various molecules and signaling pathways jointly initiated the development of tumor. For example, preclinical data showed that the PI3K pathway is not activated in ICC, 50 highlighting its necessity for the transformation process.

## Therapies for KIT Mutation and Advances in Research

Imatinib mesylate (IM) is the cardinal therapy for most GIST patients with KIT mutations in the advanced phase 6 ; it is generally well tolerated and serious adverse effects such as interstitial pneumonia or hepatotoxicity rarely occur, in which case other options such as sunitinib3 or nilotinib in GIST with KIT exon 11 mutations may be considered. 7 51 Except for patients with KIT exon 9 mutations, the starting dose of IM is 400 mg daily for all advanced patients. 7 52 Clinical data showed that up to 5% of patients had a complete response, 40 to 68% had a partial response, and 14 to 32% had stable disease. 7 53 54 Tumors that initially progress on IM are those lacking KIT or PDGFRA mutations and those with PDGFRAD842V mutation. 52 55 56 Compared with variants carrying activating exon 11 mutations, the inhibitory activity of IM on GISTs with exon 9-mutated KIT is less effective, and further study revealed that phosphorylation of KIT was not eliminated by the treatment with IM in these patients; therefore, the downstream AKT and MAPK pathways are still persistently activated. 57 In advanced disease treated with IM, the progression-free survival (PFS) for GIST patients with KIT exon 11 mutation is typically more than 24 months, whereas for those with KIT exon 9 mutations is shorter, with 12.6 to 16.7 months. 7 52 56 58 In a randomized trial reported in 2023, the results showed that compared with 1 year of IM, 3 years of IM adjuvant therapy dramatically reduces the risk of death and improves 10-year overall survival in patients with KIT exon 11 deletion/indel mutation. 59 Studies have shown that for advanced GIST with KIT exon 9 mutations, an increased dose of 400 mg twice daily has demonstrated improved PFS. 52 56 It is important to note, however, that the dose of IM 800 mg daily has not been tested in a prospective trial in the adjuvant phase and is therefore not recommended in this setting. 7 60 61

Primary and secondary resistance to IM may occur in GIST patients. The primary resistance is related to specific tumor genotype of the primary mutation, whereas secondary resistance is associated with the development of new mutations that arise during treatment. 62 KIT secondary mutations are generally located in exons 13, 14, 17, and 18. In fact, several mutations could occur simultaneously. Based on the sensitivity of the method, secondary mutations have been found in 44 to 90% of GISTs harboring primary mutations. 63 These mutations reduce or prevent imatinib binding, by disrupting H-bonds or modifying the conformation of the protein, thus making the tumor resistant to IM first-line therapy. 64 In patients with unknown KIT mutational status, an alternative second-line treatment is sunitinib, which is a multitargeted tyrosine kinase inhibitor (TKI), 65 it is also regarded as the standard second-line therapy for secondary resistant GISTs. 40 The site of the mutation determines the response rate to sunitinib. The median PFS of patients with exon 9-mutated and exon 11-mutated GIST treated with sunitinib achieved to 12.3 and 7.0 months, respectively, 66 67 and another report showed that GISTs carrying KITAY502-3 mutations at exon 9 exhibit the highest sensitivity to sunitinib. 68 In addition, new mutations present in the KIT activation loop (mainly in exon 17) were found to be resistant to sunitinib in preclinical studies. 68 69 In an open-label, multicenter, phase II trial (NCT00137449), George and coworkers investigated a different scheme of sunitinib administration to ameliorate safety and tolerance. 39

Avapritinib is a highly effective and selective inhibitor of KIT mutants, 70 71 the starting dose and the maximum tolerated dose were 300 and 400 mg daily, which is defined by Phase I trial. 72 Its common side effects are similar to those of IM, but neurocognitive adverse reactions could also occur, due to the ability to cross the blood–brain barrier. 72 Another inhibitor, ripretinib, locks KIT in an inactive conformation. 73 In preclinical testing, the agent inhibited WT as well as KIT single and double mutants. 73 Of particular interest is its activity against cells with several types of mutation in KIT exon 17, as well as cells with dual mutations on exon 9 and exon 13, exon 9 and exon 14, exon 9 and exon 17 that are not well treatable with currently available agents. In an ongoing phase I trial, no clear maximum tolerated dose was identified. 74 Janku et al has reported the results of the first in-human phase I study of ripretinib, in their research, 150 mg once daily was established as the recommended phase II dose (RP2D), the objective response rate (ORR) is 11.3%, with a range of 7.2 to 19.4%. 74 Moreover, median PFS ranging from 5.5 to 10.7 months were observed. 74 These data suggested that ripretinib has a favorable safety profile and substantial promising efficacy in advanced GIST patients refractory to approved agents.

Up to now, great progress has also been made in the study of related molecular mechanisms. Transmembrane glycoprotein Endoglin (ENG), involved in transforming growth factor receptor system, has been found in overexpressed in both human and mouse model of GISTs 75 ; Gromova et al propound that increased ENG expression in KIT-mutated GISTs is indirectly mediated by DNA hypomethylation, but the underlying mechanism of regulation on DNA methylation remains undefined. 76 Lemur tyrosine kinase-3 (LMTK3) is a crucial player in regulation of genes transcription, translation, and the stability of proteins; it is also closely implicated in tumorigenesis promoting. 77 By accelerating the translation rate of the KIT gene, LMTK3-mediated secondary mutations that contributed to resistance to IM. 78 Hedgehog pathway influenced the level of KIT mRNA via glioma-associated oncogene homolog isoform 1, 2, 3. 79 Besides, the Hedgehog pathway was discovered to has crosstalk with signal cascades of PI3K/AKT/mTOR and RAF/MAPK/ERK, which are involved in the KIT regulation as well. 3 Importantly, in vivo experiment showed that targeting the Hedgehog pathway can reenhance the sensitivity of GIST cells to TKIs. 3

PI3K pathway is the dominant signal directly engaged by mutant KIT oncogenic cascade in GIST and is associated with IM resistance. 17 50 Therefore, the clinical effect of PI3K inhibitor combined with IM in the treatment of GISTs is still under further evaluation. 80 Suppression of ACK1 markedly inhibits cell migration both in IM sensitive and resistant GIST cell lines, which is associated with downregulation of PI3K/AKT/mTOR and RAF/MAPK signaling pathways. 81 A recent study further sheds light on the role of PI3K in the immunotherapy of GISTs; the researchers found that PD-1/PD-L1 blockade reduced the apoptosis of CD8 ^+^ T cells by the PI3K/Akt/mTOR signaling pathway. 49 The fibroblast growth factors (FGFs) signal pathway has an important effect on various cell physiological processes such as cell proliferation, survival, and migration. Its dysregulation is extensively involved in several types of cancers. 3 Highly expressed FGF2 in GISTs was also believed to be associated with IM resistance. 82 After binding to its receptors FGFR, FGF2 induces the reactivation of KIT and MAPK pathways. 83 Moreover, compared with single V558Δ KIT mutation, mice with the V558Δ; V653A mutant displayed enhanced activation of STAT3 and STAT5 due to mislocalization of Golgi and contributing to the increased tumor oncogenesis. 84 Adenosine monophosphate deaminases 3 (AMPD3) is a main catalyzer in nucleotide metabolism and energy balance in cells and reported to be significantly related to KIT expression in GIST. After treatment of siRNAs targeting either KIT or AMPD3, both expression of them were comparably inhibited, indicating that KIT and AMPD3 may form a positive feedback loop to promote their reciprocal expression. 85 However, the underlying mechanism remains unknown.

As a unique marker of digestive mesenchyme immaturity, limb expression 1 (LIX1) regulates mesenchymal progenitor proliferation and differentiation by controlling the Hippo effector Yes-associated protein 1 (YAP1). 86 The activity of these two molecules is inhibited in GISTs, which is related to the expression of KIT. 87 Moreover, MAPK signaling pathway is proved to be a downstream of LIX1. 88 Upon the condition of hypoxia during IM treatment in GIST, hypoxia inducible factor 1 alpha (HIF-1α) can upregulate the transcription level of MET gene, 89 causes further activation of the downstream of MAPK, and then stabilizes ETV1 for promoting KIT expression. 90 The reactivation of MAPK by bypass signal may be one of the important reasons that therapies targeting KIT expression could not obtain satisfactory effects.

In our previous work, 897 differentially expressed genes were revealed by using RNA sequencing (RNA-seq) between IM-sensitive and IM-resistant GIST cell lines, and further investigation indicated that COL4A1, FABP4, and RGS4 may play a potential role in the clinical treatment of IM resistance in GIST. 91

During the drug screening process, it is discovered that GIST cells are high sensitivity to transcriptional inhibitors, and the mechanism is associated with the function of these compounds on the continuous expression of KIT in GISTs. For example, mithramycin A induces apoptosis by inhibits the TF, SP1, which is a major transcriptional activation of the KIT gene. 92 So, it is plausible to target KIT abnormal regulatory circus, together with kinase activity-inhibition in GIST treatment.

## PDGFRA Mutations

### Types of Genetic Mutation

PDGFRA mutations is the second most common molecular subtype of GISTs, and its incidence is less than three cases per 10 ^6^ individuals per year, and more than 90% of this subtype of GISTs are originated mainly in the stomach or the omentum, 30 with rare cases originating in the intestine or mesentery. 40 In patients with PDGFRA mutations, the proportion of males is around 58.3 to 70%, 28 93 the patients have epithelioid or mixed histological subtypes. 19 Early studies identified their association with more indolent and low-risk disease. 28 93

PDGFRA belongs to the subfamily of Type III receptor tyrosine kinases, and its mutations disrupt the receptor tyrosine kinase autoinhibitory regions, thereby resulting in a ligand-independent activation. 28 40 Variations in expression of at least 70 genes between PDGFRA- and KIT-mutated GISTs has been reported, 94 and research has shown that PDGFRA-mutated GISTs displayed higher expressions of genes associated with T-cell receptor signaling and lower expressions of genes related to AKT/PI3K pathway when compared with KIT-mutated GISTs.

In addition, compared with KIT-mutated GISTs, PDGFRA-mutated tumors are significantly more often very low/low risk, more often had tumors in the stomach and more frequently had <5 mitoses per 50 high-power field. 95 Within this cohort, low mitotic rate and gastric primary correlated with significant increases in the 5-year recurrence-free survival. 95 In a large European retrospective cohort in which 3,510 patients were enrolled, 382 patients (11%) were found with PDGFRA mutations, among them only 12.5% of these patients having metastatic disease. 55 In another large study, researchers found that GIST patients harboring PDGFRA mutations had a dramatically better disease-free survival compared with those with tumors carrying KIT mutations. 32

PDGFRA mutations are discovered in exon 18, exon 12, exon 14, and exon 4, 28 involving the A-loop encoded by exon 18, JM region encoded by exon 12 or the ATP-binding domain encoded by exon 14. 96 Mutations in exon 18 D842V located within the kinase domain activation loop is the most common PDGFRA mutation and takes up about 65% of all PDGFRA mutations in GIST. 28 30 40 Exon 12 PDGFRA mutation is more frequently detected as a deletion than a duplication, and the most frequent site is 1821T → A, causing the V561D substitution at the protein level. Otherwise, exon 14 mutation induces N659K substitution in protein, this mutation is relatively rare compared with others and is associated with a better clinical outcome. 97 In 2023, germline PDGFRA exon 15 p.G680R mutation was founded in a 58-year-old patient who presented with a gastric GIST and numerous small intestinal IFPs, which is previously undescribed. 98

### Therapies for PDGFRA Mutation and Advances in Research

First-line IM is recommended in patients with large tumors, in whom immediate resection is not possible. 3 65 Currently, IM can be used for patients with most PDGFRA mutations (except PDGFRA D842V). 3 25 GISTs with PDGFRA mutations in exon 12, exon 14 and exon 18 barely give resistance to IM; however, the most common subtype, GISTs bearing exon 18 D842V missense mutation are proved to be resistant to IM and other TKIs. 55 99 Actually some in vitro experiments suggested that nearly all exon 18 D842 mutants (apart from D842Y) have been shown to be IM resistant. 28 100 Corless and colleagues demonstrated that CHO cells stably transfected with PDGFRA mutants are resistant to IM, except for the D842Y that is sensitive. 39 Differential sensitivity dependent on PDGFRA mutation has also been reported in patient cohorts. 93 In addition, there are studies suggesting that the real percentage of patients with KIT/PDGFRA wild type is lower than was considered. These studies explain this by genetic testing errors and missing KIT/PDGFRA mutations. Under these conditions, IM is suggested and considered to be useful, even in patients with KIT/PDGFRA wild-type group. 25

Distortion of the kinase activation loop caused by PDGFRA D842V mutation confers resistance to IM in about 10% of primary GISTs. 27 62 With no effective treatments available, the prognosis for these patients is particularly dire, 7 70 and the overall survival (OS) was only 14.7 months. 55 In a study from Cassier et al, no clinical response was elicited in the subgroup of patients with D842V mutation after treated with IM. 55 In a recent study, of 16 patients with D842V-mutated GIST who received IM treatment, only 2 patients had partial response, with median time to progression of 8 months. 93 By contrast, consistent with preclinical data, 100% of patients with non-D842V mutations had clinical benefit from IM. 93 The biological mechanism proposed by the authors is multiple GIST clones existing within a patient, with some harboring imatinib-sensitive mutations. These results illustrate that even with in vitro data suggesting resistance, clinically there may be some rationale in the use of IM at some point for patients with D842V mutations in the absence of a clinical trial, 93 novel TKIs or exhausting all other lines of therapy.

Avapritinib is a novel TKI identified impressive inhibition of PDGFRA mutations; significantly, it is an important new agent for patients with PDGFRA D842V tumors who have no other proven active medical therapies. It has been approved on the basis of the phase I/II trial results for the treatment of GISTs with mutations of PDGFRA exon 18 by the U.S. Food and Drug Administration in January 2020 and specifically for GISTs with the D842V mutation by the EMA in October 2020. 72 It is the first approved therapy for GISTs patients with PDGFRA D842V mutations and considered as the current international standard of care for PDGFRA D842V tumors. 28 Given the American Society of Clinical Oncology and the Connective Tissue Oncology Society presentations congruent with respect to efficacy, the final results of the Phase I trial have shown an impressive ORR and PFS with avapritinib for patients with PDGFRA exon 18 D842V mutations. 28 It is reported that avapritinib's half maximal inhibitory concentration (IC50) is over 3000 times less than IM against PDGFRA D842V mutation. 70 In a clinical trial with 56 patients of this subset of tumors, results showed that 5 patients had a complete response (9%), 44 patients had a partial response (79%), and 7 patients had stable disease at a dose of 300 mg avapritinib daily. 72 Updated results presented at the Connective Tissue Oncology Society 2018 including 231 patients, out of which 56 (24%) had exon 18 D842V-mutated GISTs, continued to demonstrate efficacy within this population. 28 In vitro results showed that both avapritinib and ripretinib are more effective than IM, whereas avapritinib is more potent than ripretinib for PDGFRA D842V mutation. 73 However, whether ripretinib will have clinical activity against PDGFRA D842V mutation remains to be observed. While avapritinib did not present any improvement over regorafenib in another randomized phase III trial with GIST patients. 101 Moreover, in total, 8.7% of patients discontinued avapritinib due to any adverse event that were similar to those of other commonly used TKIs. 28

It is reported that mutation of PDGFRA-Thr674 to isoleucine (T674I) or arginine (T674R) induces resistance to avapritinib. 102 Subsequently, a subpocket (Gα-pocket) located in the N-lobe of the kinase domain of PDGFRA and KIT is identified for the first time, this Gα-pocket is surrounded by amino acids of key regulatory elements. 103 Targeting the Gα-pocket offers great potential to impact both potency and selectivity positively and to overcome acquired resistance mutations and should be considered for the development of next-generation inhibitors. These structural findings will guide the development of next-generation inhibitors to overcome toxicity-associated brain permeability and the current obstacles of resistance mutations in GIST.

## SDH Mutations

### Types of Genetic Mutation

SDH is a mitochondrial enzyme complex and located in the inner membrane of the mitochondria, 104 it comprised of four subunits: SDHA, SDHB, SDHC, SDHD. 9 Genetic alterations in any of these four genes or SDHAF2 lead to SDH complex dysfunction and loss of SDHB expression. 105 SDH Mutations have been demonstrated to be implicated in the tumorigenesis of different types of cancers including GISTs. 104 106 Almost 50% of KIT and PDGFRA WT-GISTs are marked by alterations involving the SDH complex 40 and fall into SDH-competent or SDH-deficient. Their SDH status should be determined since some SDH-competent GISTs are aggressive and tend to metastasize, whereas SDH-deficient tumors are characterized by an indolent overall clinical course and longer OS, although they do not respond to systemic therapies. 9 SDH-competent GISTs were mainly detected in older patients and 82% of all cases located in the small bowel, whereas SDH-deficient tumors arise almost exclusively in the stomach. 104 107 SDH-deficient GISTs are frequently accompanied by early lymphovascular invasion and consequent involvement of the lymph nodes and less involved in the liver. 104 This subtype of tumor mainly occur in children, adolescent, and young adults, with a predominance in females. 7 9

SDH-competent GISTs include those with mutations in genes of the RAS–MEK–MAPK pathway, those with translocations involving NTRK or FGFR genes and others with very rare mutations. 7 9 SDH-deficient GISTs include those with mutations in genes encoding SDH subunits and those with epigenetic suppression of SDH expression. The loss of SDH activity has important consequences for the pathogenesis of these tumors. 108 109 Approximately a half of SDH-deficient GISTs are related to hypermethylation of the SDHC promoter, which cause decrease of SDHC proteins, germline mutations in SDHA occur in around 30%, whereas those in SDHB, SDHC, and SDHD are less frequent. 110 SDH-deficient GISTs exhibit specific clinical features and pathological characteristics, are commonly multifocal, and often associated with metastatic disease. Furthermore, they often show a lobulated and multinodular growth pattern, an epithelioid phenotype, and a common lymphovascular invasion. 97 106 111 Immunohistochemical negative detection for SDHB is a diagnostic marker of SDH-deficient tumors. 104 Besides, theses tumors are found to be uniformly immunohistochemically positive for both KIT and DOG1/Anoctamin-1. 106 A de novo dedifferentiated GIST with the SDH deficiency was reported recently, the SDHB staining of tissues from this 32-year-old Chinese woman was negative, the next-generation sequencing analysis showed the SDHC mutation and the MDM2 amplification was only found in the spindle cell area. 112

How the dysfunction of SDH leads to GISTs? One of the hypotheses proposed that the mechanisms are associated with the activation of pseudohypoxia pathway. 104 SDH deficiency lead to succinate accumulation, which inhibits propyl hydroxylases resulting in induction of the hypoxic response in normoxic conditions. 104 113 Then, hydroxylation of HIF-1 is suppressed leading to a decrease in degradation; subsequently, they translocated to the nucleus and participates in important biological processes such as angiogenesis, cell proliferation, and glycolysis by regulating the expression of multiple genes. 113 114 The changes of these molecule contributes to the transformation of normal ICC into SDH-deficient GIST. 7 This vies was further supported by additional studies. 104 113 The ten-eleven translocation family of DNA hydroxylases is also inhibited by accumulated succinate, resulting in the genome-wide DNA hypermethylation detected in SDH-deficient GIST. 7

Additionally, the accumulation of reactive oxygen species, which are mainly produced in complex I and complex III in ETC is reported to has important consequences for the loss of function of the SDH, their relationship is also considered to be implicated in tumor pathogenesis. 115 Recently, some scholars have proposed that SDH knockdown increases intracellular levels of succinate, by which a-KG dependent dioxygenases, JIp1, which is involved in sulfur metabolism and Jhd1, which belongs to the JmjC-domain containing histone demethylase enzymes were inhibited. That could lead to tumor formation by causing epigenetic changes. 104 116 The level of insulin-like growth factor 1 receptor (IGF1R) has been reported to be particularly enhanced in SDH-deficient GISTs 106 117 and inhibitor of IGF1R can induces apoptosis in SDH-deficient tumors via suppressing the downstream signaling pathways such as MAPK and PI3K/AKT. 31 36 Moreover, SDH-deficient GISTs display a depletion of immune competence, suggesting that this GIST subgroup can be considered a noninflamed tumor. 118 In a recent study, the researchers discovered that MGMT promoter methylation was significantly elevated and MGMT expression dramatically decreased in SDH-deficient GISTs compared with TK mutant or SDH preserved GISTs, but no correlation was found between SDH subunit gene mutations and MGMT methylation levels. 119 As SDH-competent GIST are often less responsive or not responsive to currently approved TKIs but may respond to other therapies, such as NTRK or BRAF inhibitors, identifying these mutations may help determine the appropriate treatment. 120

### Therapies for SDH Mutation and Advances in Research

To date, the medical management of SDH-deficient GISTs is still controversial because of limited data available, both due to the rarity of this molecular subset of GIST and to the lack of SDH deficiency characterization in most studies. 110 The mortality is almost 15%, although these tumors are unpredictable since metastasis of cancer cells may be initiated after a long time. 40 121

In SDH-deficiency syndromes, the recommendations for treatment and monitoring are different. 46 In most cases, these patients are part of clinical trials or their treatment takes place in tertiary care centers. 122 There are data suggesting that surgical resection may not be beneficial for some patients with WT-GISTs. 25 Furthermore, SDH-deficient tumors are frequently resistant to TKIs, normally used in patients with advanced GISTs and KIT/PDGFRA mutation. This can be explained by the absence of gain-of-function tyrosine kinase mutation. However, although limited efficiency of these therapeutic agents is demonstrated, some patients with SDH-deficient GISTs may benefit from this treatment. 25

Despite the frequent occurrence of lymph node and hepatic metastases, the disease course of SDH-deficient GISTs is often clinically indolent, pointing to the need for careful selection of therapy or watch-and-wait strategies in advanced disease. 7 9 Generally, SDH-deficient GISTs are widely considered not sensitive to TKIs, 18 as all other KIT/PDGFRA WT GIST. Thus, there is a consensus to avoid IM or any adjuvant treatment in this rare molecular subset of GIST. 123 Recent advances on the molecular background of SDH-deficient GISTs have shifted the therapy focus from the standard TKIs to other therapeutic strategies.

SDH-deficient tumors have a slow evolution, the therapeutic management of these patients is not yet clearly established. They usually do not respond to IM treatment but may have a response to sunitinib or regorafenib and may be candidates for various clinical trials. 25 124 Sunitinib has activity against in SDH-deficient tumors, possibly owing to inhibitory activity against VEGFR. 7 The toxicity profile of sunitinib includes diarrhea, fatigue, hypertension and cardiac toxic effects, hypothyroidism, and hand–foot syndrome. 7 125 With the combination of BGJ398 and sunitinib, SDH-GIST patients may get better outcomes. 126

GIST with SDH deficiency may be partly sensitive to VEGFR2 inhibitors, such as regorafenib and sunitinib. 7 As previously mentioned, IGF1R was overexpressed in SDH-deficient GISTs, suggesting a potential role of IGF1R as a target for inhibition therapy. 109 The oral IGF-1R TKI linsitinib has been tested in a phase II study on adult and pediatric patients with WT GIST, including 15 SDH-deficient GISTs, and linsitinib yielded stable disease in 40 and 52%, respectively, of patients at 9 months, 77 suggesting a potential benefit of linsitinib in this patient population. 127 Recently, in a phase II trial, vandetanib has been evaluated in patients with SDH-deficient GISTs. Unfortunately, no partial or complete responses have been obtained, indicating that vandetanib is neither effective nor well tolerated in these patients. 128

Regorafenib is an oral multikinase inhibitor and its clinical efficacy needs to be further evaluated. Its activity has been confirmed in a phase III trial with advanced GIST patients progressing to IM and sunitinib 129 as well as in SDH-deficient GISTs: two patients had a partial response and four patients had stable disease. 130

A phase II trial with the cooperation of Spanish, French, and Italian sarcoma groups showed that 60% WT-GIST patients experienced some tumor shrinkage after received regorafenib, with partial responses and stabilization observed in 13 and 87%, respectively. Importantly, SDH-deficient GIST showed better clinical outcome than other WT-GIST. 131 Taken together, the previous information indicates that regorafenib may be more advantageous than IM for advanced WT-GIST patients as upfront therapy.

Since promoter methylation is widespread in SDH-deficient GISTs, alkylating agents may have a potential role in this tumor subgroup. 132 A phase II trial on temozolomide in advanced SDH-GISTs is still ongoing; a prolonged disease stability after 18 consecutive cycles of temozolomide has been recently reported in a female metastatic and progressive SDH-deficient GIST. 123

### BRAF/NF1 Mutations and Advances in Research

GISTs with mutations in BRAF and NF-1 are usually found in older patients and they have more aggressive disease. 133 134 The BRAF gene codes for a serine/threonine protein kinase that is involved in cell cycle regulation and carcinogenic modulation of cell response to growth signals. 27 40 It is a crucial player in tumorigenesis, known as the most deregulated genes among different types of cancer. 135 GIST with BRAF mutations also arise in in the small intestine and show spindle cell morphology. 7 136 Patients with these tumors have variable prognostic outcomes. 7 127 The occurrence of BRAF (V600E) mutation was originally described by Agaram and colleagues in subgroups of WT and IM-resistant GISTs. 137 Initially, BRAF and KIT/PDGFRA mutations were considered to be mutually exclusive, but recently, the BRAF mutation is found in 2% of GISTs patients carrying mutated KIT/PDGFRA in several studies, and these tumors are resistant to IM, 138 highlighting the possibility that the frequency of BRAF coexistence with KIT/PDGFRA mutations was underestimated in past years. In a recent study, the concomitant occurrence of BRAF/KIT and BRAF/PDGFRA mutations in GISTs is confirmed by using a quantitative competitive allele-specific Taq-Man duplex polymerase chain reaction. 139 Accordingly, two spindle cell phenotype GIST cases harboring novel BRAF fusion genes arising in two young–adult women in the small bowel and esophagus have been reported. In both cases, immunohistochemical analysis revealed a diffuse reactivity for DOG1, whereas KIT was weakly positive or negative. Conversely, targeted RNA-seq with Archer Fusion Plex revealed the occurrence of a fusion between BRAF with either AGAP3 or MKRN1 gene partners. 140 As an uncommon but established oncogenic driver in GISTs, the importance of BRAF mutation is gradually realized by researchers, and further investigation on its role as a target marker for TKIs is needed. These BRAF-mutated GISTs are resistant to IM but may be sensitive to BRAF inhibitors such as dabrafenib and MEK inhibitors. 7 Gowda et al recorded the treatment process of a GIST patient with a BRAF V600E mutation, who is a 67-year-old woman diagnosed with high-risk tumor following initial resection. 141 After initially treated with IM for 7 months, she was started on sunitinib and subsequently regorafenib, which were both discontinued. 141 Then, dabrafenib was used based on the presence of a BRAF V600E mutation, and the patient was in stable condition for 19 months. 141 Afterward, her disease continued to progress and several of other medications did not achieve the desired effect. 141 GIST tumors with other mutations in RAS genes or PIK3CA or with gene fusions involving NTRK3 or FGFR1 are very rare but require molecular identification in case of relapse, as specific treatments targeting activated NTRKs and FGFRs, such as larotrectinib, entrectinib, or erdafitinib are now available. 9 23

NF1 is a tumor suppressor gene and encodes neurofibromin, a negative regulator of RAS proteins. Biallelic inactivation of NF1 may induce tumor formation, and 7% of patients with NF1 loss develop GISTs. 7 142 143 GISTs with this rare subtype of mutations often located in small intestine and metastatic, nevertheless, have low mitotic rate and are associated to a good prognosis, 144 145 frequently multiple and typically lacking PDGFRA and KIT mutations. 142 However, somatic NF1 inactivation has also been reported in KIT-mutated GIST. 146 147 Research showed that NF1-mutated GISTs without KIT/PDGFRA mutations are resistant to currently approved TKIs. 142 Three patients with NF1 mutation was reported to show synchronous ampullary neuroendocrine tumor (NET) and GIST, which is extremely rare. 148 After surgical resection, there was no recurrence during the postoperative follow-up period of 10, 9, and 2.7 years. 148 The possible coexistence of other tumors in NF1 patients is relatively higher than that in the general population, but both NETs and GISTs occurring in NF1 patients tend to be smaller in size.

## Conclusions and Perspectives

In this review, we have collected data from the literature in order to present the current update of four major mutations occurring in GISTs and summarize the current treatment and clinic trials for different types of GISTs. Moreover, we introduced the advances in research of GISTs harboring different mutations. It has become increasingly clear that GIST with different mutation has unique biological and clinical characteristics, and the responses to treatments are significantly influenced by the underlying genotype of the disease.

KIT and PDGFRA mutations are the two major types of GISTs; SDH-mutated GISTs have also received extensive attention. BRAF/NF1 mutations are relatively rare in GISTs, and related research is very limited. As the sensitivity of detection methods increases, more and more rare mutations may appear, and new types of mutations may be discovered. GIST is paradigmatic models of cancers benefiting from personalized medicine approaches with TKIs. Considerable progress has been made in the routine management of patients with GIST over the last two decades, mainly due to the discovery of oncogenic drivers and the identification of predictive biomarkers and targeted drugs useful for precision medicine. However, current TKI-based therapies do not satisfy long-term disease control once the disease develops resistance, or because some GIST subtypes do not respond.

Hence, further research should focus on new targets and drugs. The next phase of clinical research could focus on identifying new therapeutic targets. In addition, addressing secondary drug resistance to IM has been the key to improving prognosis in GIST patients. According to the current research achievements, combined inhibition of drug resistance mechanisms with IM therapy and combined inhibition of multiple drug resistance mechanisms are anticipated to become new strategies for the treatment of GISTs. Over the past few decades, many important discoveries have been made in research of GISTs, and it is expected that scholars will further reveal the mechanism of tumor occurrence and drug resistance, so as to guide the development of new drugs and the formulation of treatment strategies.
